# Gene editing in livestock: innovations and applications

**DOI:** 10.1590/1984-3143-AR2024-0054

**Published:** 2024-09-23

**Authors:** Paula Rodriguez-Villamil, Benjamin Paul Beaton, Rebecca Lynn Krisher

**Affiliations:** 1 Research and Development Genus PLC., Deforest, WI, USA

**Keywords:** gene-editing, livestock, production, biomodels, welfare

## Abstract

Gene editing technologies have revolutionized the field of livestock breeding, offering unprecedented opportunities to enhance animal welfare, productivity, and sustainability. This paper provides a comprehensive review of recent innovations and applications of gene editing in livestock, exploring the diverse applications of gene editing in livestock breeding, as well as the regulatory and ethical considerations, and the current challenges and prospects of the technology in the industry. Overall, this review underscores the transformative potential of gene editing in livestock breeding and its pivotal role in shaping the future of agriculture and biomedicine.

## Introduction

In animal production, gene editing tools represent a significant improvement, overcoming the limitations of traditional breeding methods. While conventional approaches like natural breeding and trait selection have historically driven progress, they are often yield unpredictable or inefficient outcomes. Traditional breeding struggles to introduce or enhance high-quality genes without inadvertently introducing undesirable traits, such as infertility or low growth (Bishop and Van Eenennaam, 2020). Furthermore, continued genetic improvements through selective breeding rely on the existence of beneficial natural genetic variation within the population. Without such variation, there is limited scope for trait improvement (Whitworth et al., 2022; Menchaca, 2020). In contrast, gene editing offers a novel pathway towards rapid advancement, characterized by precision and efficiency.

Furthermore, it’s crucial to address the evolving requirements of the global food production, including environmental, economic, and social concerns (Henchion et al., 2021). The FAO projects a continued increase in the consumption of animal protein, highlighting the need for higher-quality foods to meet global demands (FAO, 2009). Achieving this necessitates both genetic and sustainable management-based productivity enhancements (Fahrenkrug et al., 2010). Thus, this technology presents opportunities to accelerate genetic improvement with unprecedented accuracy, revolutionizing agricultural productivity, sustainability, and animal welfare simultaneously (Mueller and Van Eenennaam, 2022: Mariano et al., 2024).

In recent years, changing public opinion and regulatory landscapes have permitted the integration of gene-edited animals into production systems and even for human consumption (Epstein et al,2021). Programs like the FDA/CVM in the USA regulate and evaluate the risk of intentional genomic alterations (IGAs) in animals before they enter the food supply or the environment. Several models, including Atlantic salmon, the α-gal pig, and the SLICK cattle, have been approved for human consumption through such programs. The use of safer and more efficient technologies has demonstrated that genetically modified animals serve purposes beyond food, contributing to human health, serving as bio-models for disease treatment, and providing biomaterials for tissue and organ reconstruction (Kues and Niemann, 2004).

## Gene editing tools

Several gene editing tools are utilized in the production of livestock animals, primarily involving the generation of a double-strand DNA break followed by repair through non-homologous end joining (NHEJ) or homology directed repair (HDR) pathways. These tools include Zinc-Finger Nucleases (ZFNs), Transcription activator-like effector nucleases (TALENS), CRISPR-associated 9 (Cas9) system (Carlson et al, 2012; Navarro-Serna et al., 2020).

### Zinc Finger nucleases

Zinc Finger nucleases were among the earliest technologies developed to induce site-directed DSBs at specific loci by anchoring endonuclease catalytic domains to modular DNA-binding proteins. ZFNs, are chimeric proteins that combine zinc finger proteins with endonucleases, usually the cleavage domain of the FokI restriction enzyme (Kim and Kim, 2014). ZFNs constitute an effective tool to perform gene silencing (knockout), correcting defective genes, or to introduce DNA sequences at DSB sites (Carroll, 2011). ZFNs can be introduced into cells or embryos, as DNA, RNA, or protein (Harrison et al., 2014).

This tool has been utilized effectively in editing the genomes of various livestock animals, including fish (Dong et al., 2011), cattle (Liu et al., 2014), and pigs (Bao et al., 2014; Qian et al., 2015). However, concerns have been raised regarding its off-target activity (Pattanayak et al., 2011). Additionally, the cost of effective commercial Zinc Finger Nuclease (ZFN) reagents remains high, and despite the availability of some open-source libraries for the project, the process of engineering ZFNs remains complex.

### TALENs

Transcription activator-like effector nucleases (TALENs) are naturally produced by phytopathogenic bacteria of the genus Xanthomonas sp. and naturally function to modulate host gene expression. After delivery to host cells via the bacterial type III secretion system, TAL effectors enter the nucleus, bind to specific effector sequences in host gene promoters, and activate transcription (Bogdanove et al., 2010). As a DNA editing tool, TAL effectors are fused to the catalytic domain of the FokI nuclease to create DSBs at the target DNA (Miller et al., 2011). TALENs function in pairs, binding opposite targets through a spacer, which brings the FokI domains together, working as a dimer, to cleave the DNA creating the DSB (Cermak et al., 2011).

Compared to ZFNs, TALENs have higher specificity and are easier to design and construct. Consequently, TALENs have been successfully deployed for genome editing of livestock animals such as pigs (Carlson et al., 2012), cattle (Carlson et al., 2012), sheep (Li et al., 2016), and goats (Cui et al., 2015). However, TALENs have been diminishing use with the emergence of the CRISPR/Cas9 system.

### CRISPR

The CRISPR/Cas9 system, originally an adaptive immune system in bacteria to protect against invading viruses, has been repurposed for gene editing, offering a precise and versatile tool for targeted DNA modification(s) (Doudna and Charpentier 2014; Hsu et al., 2013). It utilizes a single guide RNA (sgRNA) of 20 nucleotides that undergoes Watson-Crick base pairing with a specific DNA sequence adjacent to a protospacer adjacent motif (PAM: NGG, where N is any of the four bases of DNA) sequence, guiding the Cas9 endonuclease to induce a DSB at the target DNA sequence (Cong et al., 2013). Unlike previous technologies like ZFNs or TALENs, which require custom protein engineering for each target sequence, CRISPR/Cas9 relies solely on the design of the sgRNA for specificity (Doudna and Charpentier, 2014).

Following cleavage, the cell’s repair mechanisms come into action. The nonhomologous repair pathway (NHEJ) becomes the cells preferred path of choice, albeit error-prone and can lead to the formation of random short insertions and/or deletions and thus change the structure of mutations and disrupting gene function. Alternatively, with the introduction of an exogenous repair template, homology-directed repair (HDR) can occur, allowing for precise gene editing or the insertion of desired DNA sequences. With the ease of this system and NHEJ being the cells preferred repair mechanism the NHEJ represents the main method of generating gene knockouts mediated by CRISPR/Cas9.

The NHEJ repair mechanism has led to the generation of the first successful disruption of endogenous genes in a variety of livestock species (Whitworth et al., 2014; Ni et al., 2014; Wang et al., 2015; Gao et al., 2017). Similarly, this technology has successfully produced livestock knock-in animals by HDR like pigs (Wang et al., 2018) and small ruminants (Eaton et al., 2019), and has been widely used until today. This tool has been widely studied and applied to improve livestock heredity, reproduction, and nutrition levels. All the CRISPR and CRISPR-associated protein (Cas) can be easily customized to effectively introduce mutations at specific locations within genes in mammalian cells (Cong et al., 2013).

Recent developments in CRISPR technology are presenting new promising CRISPR variants. For example, base editing (BE), a variation of Cas9 that consists of a catalytically impaired Cas9 endonuclease fused to a reverse transcriptase allowing precise targeted insertions, deletions and point mutations without requiring DSBs or donor DNA templates (Perisse et al., 2021). BE systems offer lower off target activity and fewer by products than previous alternatives (Anzalone et al., 2019). Based on these advantages, several groups have produced various models to improve livestock production, reproduction, milk-production, and wool-production traits (Li et al., 2019; Zhu et al., 2022: Wang et al., 2022).

## Delivery methods

The development of gene-editing techniques in livestock animals has increased in recent years, particularly with the advancements in technologies such as CRISPR/Cas9 and improvements in delivery methods. Although the first GE livestock animals were produced by micromanipulation in 1985 (Hammer et al., 1985), the conventional gene targeting approaches of that time were inefficient and the techniques were limited in scope. However, over the past 20 years, the emergence of new tools has demonstrated relevant techniques for producing different gene-edited livestock models across various species.

### Zygote editing

Editing zygotes, as opposed to other methods such as somatic cell nuclear transfer (SCNT), offers advantages for production purposes, allowing for the creation of diverse foundation animals that are genetically distinct, as opposed to identical animals derived from a clonal cell line (Bishop and Van Eenennaam, 2020; Mariano et al., 2024). Initially, microinjection was the traditional method used to deliver gene editing reagents. However, newer techniques like electroporation have emerged as promising alternatives, offering a less time-consuming and more cost-effective approach.

**Figure 1:**
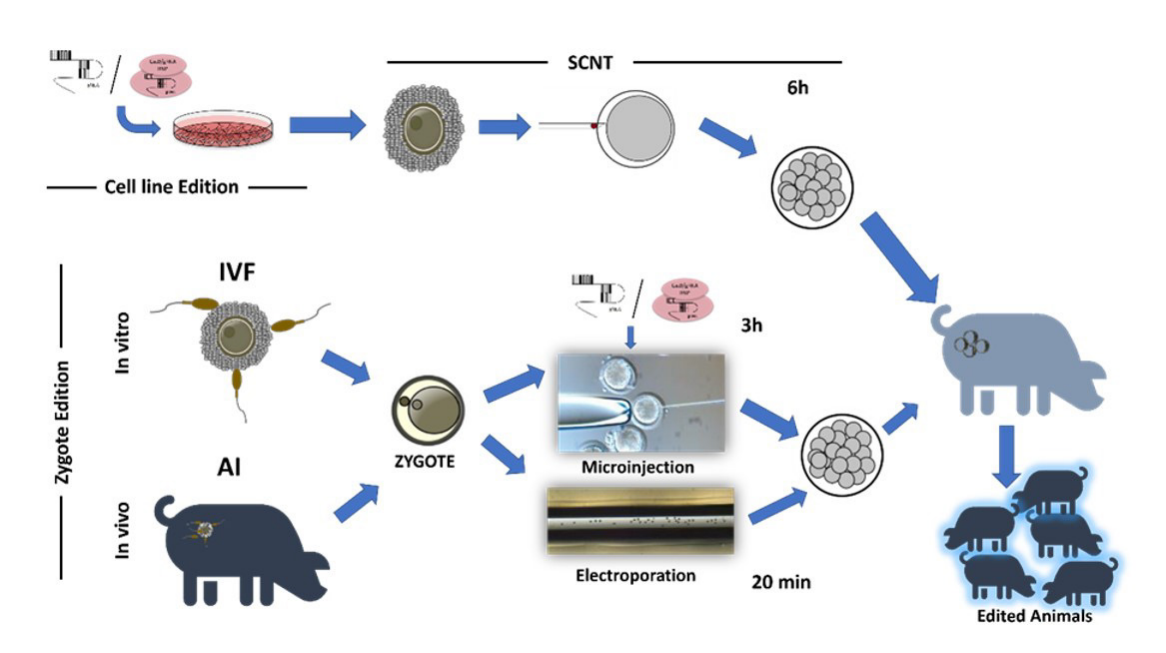
Schematic diagram of gene editing delivery methods to produced livestock animals. IVF= in vitro fertilization, AI= Artificial Insemination, SCNT=Somatic cells nuclear transfer.

Microinjection involves injection of gene editing reagents by micromanipulation of one-cell stage embryos (Figure 1). Initially, microinjection proved to be effective in producing several transgenic species such as mice, rabbits, pigs, sheep, cattle, and goats through microinjection of gene constructs into the pronucleus of a zygote (Wall, 1996). With the use of CRISPR/Cas9, the process became easier and faster, allowing the microinjection into the cytoplasm rather than the pronucleus. Thus, CRISPR/Cas9 microinjection has become more innocuous and efficient in livestock animals (Menchaca et al., 2020).

Furthermore, the method requires the use of expensive equipment and skilled personnel to microinject zygotes with genome-editing reagents in a single zygote cadence. In addition to these limitations, the timing between microinjection and fertilization significantly impact the results, sometimes making it a challenge to achieve consistent results. It is known that to obtain efficient and consistent results, it is crucial to attempt the narrow time window between gamete fusion and the first embryo cell division for delivering editing tools. Thus, many models prefer, when possible, in respect to species, to use in vitro fertilized embryos to determine the most appropriate time to deliver gene editing reagents prior to the first cell division and avoid mosaicism (Lin et al., 2021; Ratner et al., 2021).

Electroporation of zygotes offers a simplified and streamlined approach for transfecting mammalian zygotes (Figure 1). The technique was initially used for gene transfer and introducing other agents into cells (Knutson and Ye, 1987). However, in the last decade, the success of the technique to introduce genes into zygotes of different species has been demonstrated. The first protocol was established in pre-implantation embryos in mice (Peng et al., 2012), followed by various protocols for editing zygotes by electroporation (Qin et al., 2015; Chen et al., 2016) enhancing the system efficiency. However, none of these protocols were repeatable in other species such as bovine (Wei et al., 2018), largely due to differences in embryo structure, e.g. the zona pellucida. The electroporator system directs pulses of electrical currents through the zygotes via electrodes creating temporary micro-holes in the zona pellucida and plasma membrane which facilitate the movement of genome editing reagents into the zygotes (Lin et al., 2021). Modifications to the different parameters (voltage, number of pulses, and pulse length), have demonstrated to be adaptable to the needs of the different species and enabling the generation of edited animals like bovine (Camargo et al., 2020), buffalo (Punetha et al., 2024), and porcine (Tanihara et al., 2016) with success. The workflow of delivering genome-editing reagents is considerably accelerated compared to microinjection, affording simultaneous electroporation of 35 to 100 zygotes (Modzelewski et al., 2018).

The recent success of electroporation can also be attributed in part to its combination with Cas9 as a protein. The compact nature of the RNP complex allows it to easily enter through the pores generated in zygotes, contrasting with larger Cas9 mRNA or other editing tools. This makes the process more efficient and less time-consuming (Ratner et al., 2021). Furthermore, this technique is continuously evolving with new adaptations, such as the novel method called improved-Genome editing via Oviductal Nucleic Acids Delivery (i-GONAD). i-GONAD delivers CRISPR RNPs to E0.7 embryos via in situ electroporation. The oviductal electroporation effectively edits the zygotes while retaining the reproductive function of the female. This approach holds high promise as an in vivo gene therapy tool for germline gene correction (Takabayashi et al., 2018).

### Cell line editing

On the other hand, as an alternative to the zygote edition (Microinjection or electroporation), is the editing of somatic cells by transfection and the production of the animal by SCNT (Figure 1). Since the birth of Dolly in 1996 (Wilmut et al., 1997), SCNT has become the cell-mediated platform for livestock genetic engineering (Perisse et al., 2021). This technique offers several advantages, due to the efficiency of editing in somatic cells and the possibility to have multiplex editing (Sato et al., 2017). Additionality, it enables the manipulation and characterization of genetically modified cells before SCNT, ensuring the birth of animals with the desired genotype and desired sex (Clark and Whitelaw, 2003). This capability facilitates the replication of the same line of gene-edited animals and, more importantly, reduces mosaicism in animals generated (Ratner et al., 2021).

However, this technique is time consuming like microinjection and requires highly trained personnel and expensive equipment. Furthermore, it’s efficiency is hindered by the low proportion of transferred embryos and low pregnancy rates. There are also concerns about potential SCNT related epigenetic alterations and an increase in stillborn or low newborn survival rates (Keefer, 2015; Perisse et al., 2021).

## Applications

The application of gene editing technology in animals has significantly contributed to various aspects of livestock production, including the development of desirable production traits, reducing the generation interval, and improving animal resistance to diseases, making them more adapted and resilient (Mueller and Van Eenennaam, 2022; Mariano et al., 2024). Additionally, diverse approaches have demonstrated potential for biomedical applications. These include the generation of animal models for studying human genetic diseases, the production of biopharmaceuticals, and the exciting prospects of utilizing gene-edited animals as potential sources of tissues and organs for human transplantation.

## Agricultural

### Production traits

In efforts to increase animal protein production, early research focused on modifying growth hormone genes in fish. This editing led to enhanced growth rates, with fish growing 30 to 50% faster and larger than the wildtype counterparts. One notable example is the growth hormone transgenic Atlantic salmon (AquAdvantage® salmon), which contains a copy of the growth hormone from Coho salmon (Du et al., 1992). Approved by the FDA for human consumption in 2015 and for commercial production in Canada and USA in 2016, these salmon represent a milestone in genetic modification for agricultural purposes.

Another common approach to increase productivity is through the knockout of the myostatin gene (MSTN). This genetic modification allows for an increase in muscle percentage, leading to enhanced meat production across various animal species, including sheep (Crispo et al., 2015), cattle (Proudfoot et al., 2015), pigs (Tanihara et al., 2016), fish (Zhong et al., 2016; Khalil et al., 2017), and goats (Wang et al., 2018).

### Animal health

Harnessing the potential of gene editing technologies like CRISPR/Cas9 offers a transformative approach to combatting diseases in bovine and porcine populations (Ran et al., 2013; Proudfoot et al., 2015; Ruan et al., 2017). By precisely manipulating key genetic factors involved in disease susceptibility or resistance, researchers and genetic companies aim to bolster the natural defenses of these animals against prevalent pathogens. In the bovine sector, ailments such as bovine respiratory disease complex (BRDC) and bovine viral diarrhea (BVD) significantly impact productivity and profitability (Workman et al., 2023). Other models for disease resistance have been developed for common infections such as mastitis, tuberculosis, and bovine spongiform encephalophathy through the insertion of genes like human lysozyme (Liu et al., 2014), human NRAMP1 (Gao et al., 2017) and disruption of the PRNP gene (Bevacqua et al., 2016). Similarly, in porcine farming, diseases like porcine reproductive and respiratory syndrome (PRRS) and African swine fever (ASF) pose persistent challenges (Prather et al., 2017; Carriquiry et al., 2021). Through targeted genetic modifications, pigs can be fortified with enhanced immunity against these devastating diseases, safeguarding herd health and global pork production. Genus plc recently published successful development of a sizable PRRS-resistant pig population, providing compelling evidence of the practical application of genetic technologies in addressing disease and health-related traits (Burger et al., 2024; Nesbitt et al., 2024).

Gene editing holds promise in developing animals with heightened resistance to these infections, potentially reducing the need for costly interventions and pharmaceutical treatments. While ethical and regulatory considerations persist, the application of gene editing represents a paradigm shift in disease management strategies for sustainable agriculture and food security.

### Welfare

With the aim of promoting a more sustainable and welfare-conscious animal production system, gene editing has also been developed to mitigate the need for labor-intensive management practices that rely on chemical or mechanical methods. For instance, gene editing techniques such as polled horned editing in bovine to avoid the dehorning process (Carlson et al., 2016) and KISS1 knockout editing in porcine to avoid the castration process (Flórez et al., 2023), thereby protecting the welfare of animals and their handlers.

In addition, to enhance the adaptability and resilience of certain breeds into different environmental conditions, some genes can be selected from nature and introduced into non-adapted breeds. For example, the PRLR gene (Rodríguez-Villamil et al., 2021) in bovine and the UPC1gene in porcine (Zheng et al., 2017) have been targeted to improve thermotolerance. Similarly, in the case of fish, gene editing has been utilized to address issues such as biodiversity conservation and avoid the problems of bioaccumulation. By regulating reproduction, sex ratio, and even inducing sterilitity in unwanted predatory species (Matsuda et al., 2002; Karigo et al., 2014; Tang et al., 2015, Wargelius et al., 2016) gene editing offers promising solutions for promoting animal welfare and environmental sustainability in aquaculture.

## Biomedical

While the regulatory landscape for CRISPR/Cas9 in agricultural applications remain under review, gene editing in biomedicine and basic research continues to expand (Menchaca et al., 2020). Large animals are increasingly used as models in biomedicine for studying human diseases and conducting preclinical trials for testing drugs and medical devices. Porcine models are particularly prevalent due to the anatomically and physiologically similarities to humans compared to small rodents (Hou et al., 2022). Recent advances in human genomics and genetic engineering have facilitated a deeper understanding of human genetic disorders, leading to a surge in the use of pig models in studies related to cancer (Soda et al., 2007, Schook et al., 2015), diabetes (Rodriguez et al., 2020), cardiovascular diseases (Yang et al., 2011, Chen et al., 2021), immunodeficiencies (Suzuki et al., 2012) and various neurological conditions (Andersen et al., 2022). Porcine biomodels play a crucial role not only to understand human disease pathogenesis but also in facilitating the development of novel treatments, accelerating preclinical trials, enabling combinations of therapies, develop new drugs and devices, identifying new drug indications with accurate dosages, creating diagnostic tools such as biomarkers and imagine technology, and enhancing surgical intervention systems.

On the other hand, other animal models such as zebrafish are widely utilized to address significant issues in genetics, reproduction, toxicology, drug-receptor, and host-pathogen interaction. Zebrafish, being a valuable model organism for aquaculture and biomedicine applications, have been successfully employed in studies utilizing RNA interference tools, making them essential for research in various fields (Carpio and Estrada, 2006). Additionally, CRISPR/Cas9 technology has been successfully used in the development of gene modification in other non-model species as the tilapia and the atlantic salmon (Li et al., 2014)

### Xenotransplantation

Among the different porcine biomedical models, xenotransplantation represents a significant contribution. Given the imbalance between organ supply and demand for human organs, animal organs, tissues and cells are being explored as promising solutions to address the global organ shortage. However, immunological barriers pose challenges in clinical xenotransplantation. Consequently, numerous immunosuppressive therapies and gene-editing strategies, including gene knockout models, have been reported in attempts to prevent hyperacute rejection and acute vascular rejection mechanisms to promote the tolerance in pig-to-human xenotransplantation (Phelps et al., 2003; Lin et al., 2010; Estrada et al., 2015; Cowan et al., 2019)

Furthermore, recent achievements in xenotransplantation, including the first human clinical trials, have continued to spark increased interested and the development of new technologies and approaches (Mallapaty and Kozlov, 2024). Despite regulatory challenges and ethical concerns surrounding gene editing and clinical xenotransplantation, ongoing efforts are advancing new regulatory standards and fostering a more favorable public opinion about gene editing and pig models (Crane et al., 2020; Kozlov, 2022). This progress has opened avenues for novel alternatives, such as exotransplants using human stem cells for organ production through human–animal chimeras and blastocyst complementation (Wu et al., 2016).

## Conclusions

The advancements made in gene-editing present promising and safer avenues for the production and improvement of livestock animals in agriculture, addressing global requirements for enhanced production, sustainability, and animal welfare. Furthermore, these innovations provide valuable biomedical models for human research, drug development, and, notably, the potential for xenotransplantation of human cells, tissues, and organs.

## References

[B001] Andersen OM, Bøgh N, Landau AM, Pløen GG, Jensen AMG, Monti G, Ulhøi BP, Nyengaard JR, Jacobsen KR, Jørgensen MM, Holm IE, Kristensen ML, Alstrup AKO, Hansen ESS, Teunissen CE, Breidenbach L, Droescher M, Liu Y, Pedersen HS, Callesen H, Luo Y, Bolund L, Brooks DJ, Laustsen C, Small SA, Mikkelsen LF, Sørensen CB (2022). A genetically modified minipig model for Alzheimer’s disease with SORL1 haploinsufficiency. Cell Rep Med..

[B002] Anzalone AV, Randolph PB, Davis JR, Sousa AA, Koblan LW, Levy JM, Chen PJ, Wilson C, Newby GA, Raguram A, Liu DR (2019). Search-and-replace genome editing without double-strand breaks or donor DNA. Nature.

[B003] Bao L, Chen H, Jong U, Rim C, Li W, Lin X, Zhang D, Luo Q, Cui C, Huang H, Zhang Y, Xiao L, Fu Z (2014). Generation of GGTA1 biallelic knockout pigs via zinc-finger nucleases and somatic cell nuclear transfer. Sci China Life Sci.

[B004] Bevacqua RJ, Fernandez-Martín R, Savy V, Canel NG, Gismondi MI, Kues WA, Carlson DF, Fahrenkrug SC, Niemann H, Taboga OA, Ferraris S, Salamone DF (2016). Efficient edition of the bovine PRNP prion gene in somatic cells and IVF embryos using the CRISPR/Cas9 system. Theriogenology.

[B005] Bishop TF, Van Eenennaam AL (2020). Genome editing approaches to augment livestock breeding programs. J Exp Biol.

[B006] Bogdanove AJ, Schornack S, Lahaye T (2010). TAL effectors: finding plant genes for disease and defense. Curr Opin Plant Biol.

[B007] Burger BT, Beaton BP, Campbell MA, Brett BT, Rohrer MS, Plummer S, Barnes D, Jiang K, Naswa S, Lange J, Ott A, Alger E, Rincon G, Rounsley S, Betthauser J, Mtango NR, Benne JA, Hammerand J, Durfee CJ, Rotolo ML, Cameron P, Lied AM, Irby MJ, Nyer DB, Fuller CK, Gradia S, Kanner SB, Park KE, Waters J, Simpson S, Telugu BP, Salgado BC, Brandariz-Nuñez A, Rowland RRR, Culbertson M, Rice E, Cigan AM (2024). Generation of a commercial-scale founder population of porcine reproductive and respiratory syndrome virus resistant pigs using CRISPR-Cas. CRISPR J.

[B008] Camargo LSA, Owen JR, Van Eenennaam AL, Ross PJ (2020). Efficient one-step knockout by electroporation of ribonucleoproteins into zona-intact bovine embryos. Front Genet.

[B009] Carlson DF, Lancto CA, Zang B, Kim E, Walton M, Oldeschulte D, Seabury C, Sonstegard TS, Fahrenkrug SC (2016). Production of hornless dairy cattle from genome edited cell lines. Nat Biotechnol.

[B010] Carlson DF, Tan W, Lillico SG, Fahrenkug SC (2012). Efficient TALEN-Mediated Gene Knockout in Livestock. Proceedings of the National Academy of Sciences of the United States of America.

[B011] Carpio Y, Estrada MP (2006). Zebrafish as a genetic model organism. Biotecnol Apl.

[B012] Carriquiry MA, Elobeid AE, Hayes DJ, Swenson DA (2021). Impacts of An African Swine Fever Outbreak in the United States: Implications on National and Iowa Agriculture..

[B013] Carroll D (2011). Genome engineering with zinc-finger nucleases. Genetics.

[B014] Cermak T, Doyle EL, Christian M, Wang L, Zhang Y, Schmidt C, Baller JA, Somia NV, Bogdanove AJ, Voytas DF (2011). Efficient design and assembly of custom TALEN and other TAL effector-based constructs for DNA targeting. Nucleic Acids Res.

[B015] Chen J, An B, Yu B, Peng X, Yuan H, Yang Q, Chen X, Yu T, Wang L, Zhang X, Wang H, Zou X, Pang D, Ouyang H, Tang X (2021). CRISPR/Cas9-mediated knockin of human factor IX into swine factor IX locus effectively alleviates bleeding in hemophilia B pigs. Haematologica.

[B016] Chen S, Lee B, Lee AY, Modzelewski AJ, He L (2016). Highly Efficient Mouse Genome Editing by CRISPR Ribonucleoprotein Electroporation of Zygotes. J Biol Chem.

[B017] Clark J, Whitelaw B (2003). A future for transgenic livestock. Nat Rev Genet.

[B018] Cong L, Ran FA, Cox D, Lin S, Barretto R, Habib N, Hsu PD, Wu X, Jiang W, Marraffini LA, Zhang F (2013). Multiplex genome engineering using CRISPR/Cas systems. Science.

[B019] Cowan PJ, Hawthorne WJ, Nottle MB (2019). Xenogeneic transplantation and tolerance in the era of CRISPR-Cas9. Curr Opin Organ Transplant.

[B020] Crane AT, Shen FX, Brown JL, Cormack W, Ruiz-Estevez M, Voth JP, Sawai T, Hatta T, Fujita M, Low WC (2020). The american public is ready to accept human-animal chimera research. Stem Cell Reports.

[B021] Crispo M, Mulet AP, Tesson L, Barrera N, Cuadro F, Dos Santos-Neto PC, Nguyen TH, Crénéguy A, Brusselle L, Anegón I, Menchaca A (2015). Efficient generation of myostatin knock-out sheep using CRISPR/Cas9 technology and microinjection into zygotes. PLoS One.

[B022] Cui C, Song Y, Liu J, Ge H, Li Q, Huang H, Hu L, Zhu H, Jin Y, Zhang Y (2015). Gene targeting by TALEN-induced homologous recombination in goats directs production of β-lactoglobulin-free, high-human lactoferrin milk. Sci Rep.

[B023] Dong Z, Ge J, Li K, Xu Z, Liang D, Li J, Li J, Jia W, Li Y, Dong X, Cao S, Wang X, Pan J, Zhao Q (2011). Heritable targeted inactivation of myostatin gene in yellow catfish (Pelteobagrus fulvidraco) using engineered zinc finger nucleases. PLoS One.

[B024] Doudna JA, Charpentier E (2014). The new frontier of genome engineering with CRISPR-Cas9. Science.

[B025] Du SJ, Gong ZY, Fletcher GL, Shears MA, King MJ, Idler DR, Hew CL (1992). Growth enhancement in transgenic Atlantic salmon by the use of an “all fish” chimeric growth hormone gene construct. Biotechnology (N Y).

[B026] Eaton SL, Proudfoot C, Lillico SG, Skehel P, Kline RA, Hamer K, Rzechorzek NM, Clutton E, Gregson R, King T, O’Neill CA, Cooper JD, Thompson G, Whitelaw CB, Wishart TM (2019). CRISPR/ Cas9 mediated generation of an ovine model for infantile neuronal ceroid lipofuscinosis (CLN1 disease). Sci Rep.

[B027] Epstein LR, Lee SS, Miller MF, Lombardi HA (2021). CRISPR, animals, and FDA oversight: building a path to success. Proc Natl Acad Sci USA.

[B028] Estrada JL, Martens G, Li P, Adams A, Newell KA, Ford ML, Butler JR, Sidner R, Tector M, Tector J (2015). Evaluation of human and non-human primate antibody binding to pig cells lacking GGTA1/CMAH/b4GalNT2 genes. Xenotransplantation.

[B029] Fahrenkrug SC, Blake A, Carlson DF, Doran T, Van Eenennaam A, Faber D, Galli C, Gao Q, Hackett PB, Li N, Maga EA, Muir WM, Murray JD, Shi D, Stotish R, Sullivan E, Taylor JF, Walton M, Wheeler M, Whitelaw B, Glenn BP (2010). Precision genetics for complex objectives in animal agriculture. J Anim Sci.

[B030] Flórez JM, Martins K, Solin S, Bostrom JR, Rodríguez-Villamil P, Ongaratto F, Larson SA, Ganbaatar U, Coutts AW, Kern D, Murphy TW, Kim ES, Carlson DF, Huisman A, Sonstegard TS, Lents CA (2023). CRISPR/Cas9-editing of KISS1 to generate pigs with hypogonadotropic hypogonadism as a castration free trait. Front Genet.

[B031] FAO, Food and Agriculture Organization - FAO (2009). Insights from an Expert Meeting at FAO..

[B032] Gao Y, Wu H, Wang Y, Liu X, Chen L, Li Q, Cui C, Liu X, Zhang J, Zhang Y (2017). Single Cas9 nickase induced generation of NRAMP1 knockin cattle with reduced off-target effects. Genome Biol.

[B033] Hammer RE, Pursel VG, Rexroad CE, Wall RJ, Bolt DJ, Ebert KM, Palmiter RD, Brinster RL (1985). Production of transgenic rabbits, sheep and pigs by microinjection. Nature.

[B034] Harrison MM, Jenkins BV, O’Connor-Giles KM, Wildonger J (2014). A CRISPR view of development. Genes Dev.

[B035] Henchion M, Moloney AP, Hyland J, Zimmermann J, McCarthy S (2021). Review: trends for meat, milk and egg consumption for the next decades and the role played by livestock systems in the global production of proteins. Animal.

[B036] Hsu PD, Scott DA, Weinstein JA, Ran FA, Konermann S, Agarwala V, Li Y, Fine EJ, Wu X, Shalem O, Cradick TJ, Marraffini LA, Bao G, Zhang F (2013). DNA targeting specificity of RNA-guided Cas9 nucleases. Nat Biotechnol.

[B037] Karigo T, Aikawa M, Kondo C, Abe H, Kanda S, Oka Y (2014). Whole brain-pituitary in vitro preparation of the transgenic medaka (Oryzias latipes) as a tool for analyzing the differential regulatory mechanisms of LH and FSH release. Endocrinology.

[B038] Keefer CL (2015). Artificial cloning of domestic animals. Proc Natl Acad Sci USA.

[B039] Khalil K, Elayat M, Khalifa E, Daghash S, Elaswad A, Miller M, Abdelrahman H, Ye Z, Odin R, Drescher D, Vo K, Gosh K, Bugg W, Robinson D, Dunham R (2017). Generation of myostatin gene-edited channel catfish (ictalurus punctatus) via zygote injection of CRISPR/Cas9 system. Sci Rep.

[B040] Kim H, Kim JS (2014). A guide to genome engineering with programmable nucleases. Nat Rev Genet.

[B041] Knutson JC, Yee D (1987). Electroporation: parameters affecting transfer of DNA into mammalian cells. Anal Biochem.

[B042] Kozlov M (2022). Clinical trials for pig-to-human organ transplants inch closer. Nature.

[B043] Kues WA, Niemann H (2004). The contribution of farm animals to human health. Trends Biotechnol.

[B044] Li G, Zhou S, Li C, Cai B, Yu H, Ma B, Huang Y, Ding Y, Liu Y, Ding Q, He C, Zhou J, Wang Y, Zhou G, Li Y, Yan Y, Hua J, Petersen B, Jiang Y, Sonstegard T, Huang X, Chen Y, Wang X (2019). Base pair editing in goat: nonsense codon introgression into FGF5 results in longer hair. FEBS J.

[B045] Li H, Wang G, Hao Z, Zhang G, Qing Y, Liu S, Qing L, Pan W, Chen L, Liu G, Zhao R, Jia B, Zeng L, Guo J, Zhao L, Zhao H, Lv C, Xu K, Cheng W, Li H, Zhao HY, Wang W, Wei HJ (2016). Generation of biallelic knock-out sheep via gene-editing and somatic cell nuclear transfer. Sci Rep.

[B046] Li M, Yang H, Zhao J, Fang L, Shi H, Li M, Sun Y, Zhang X, Jiang D, Zhou L, Wang D (2014). Efficient and heritable gene targeting in tilapia by CRISPR/Cas9. Genetics.

[B047] Lin CC, Ezzelarab M, Hara H, Long C, Lin CW, Dorling A, Cooper DK (2010). Atorvastatin or transgenic expression of TFPI inhibits coagulation initiated by antinonGal IgG binding to porcine aortic endothelial cells. J Thromb Haemost.

[B048] Lin JC, Van Eenennaam AL (2021). Electroporation-Mediated Genome Editing of Livestock Zygotes. Front Genet.

[B049] Liu X, Wang Y, Tian Y, Yu Y, Gao M, Hu G, Su F, Pan S, Luo Y, Guo Z, Quan F, Zhang Y. (2014). Generation of mastitis resistance in cows by targeting human lysozyme gene to β-casein locus using zinc-finger nucleases. Proc Biol Sci.

[B050] Mallapaty S, Kozlov M (2024). First pig kidney transplant in a person: what it means for the future. Nature.

[B051] Mariano CG, de Oliveira VC, Ambrósio CE (2024). Gene editing in small and large animals for translational medicine: a review. Anim Reprod.

[B052] Matsuda M, Nagahama Y, Shinomiya A, Sato T, Matsuda C, Kobayashi T, Morrey CE, Shibata N, Asakawa S, Shimizu N, Hori H, Hamaguchi S, Sakaizumi M (2002). DMY is a Y-specific DM-domain gene required for male development in the medaka fish. Nature.

[B053] Menchaca A, Dos Santos-Neto PC, Mulet AP, Crispo M (2020). CRISPR in livestock: from editing to printing. Theriogenology.

[B054] Miller JC, Tan S, Qiao G, Barlow KA, Wang J, Xia DF, Meng X, Paschon DE, Leung E, Hinkley SJ, Dulay GP, Hua KL, Ankoudinova I, Cost GJ, Urnov FD, Zhang HS, Holmes MC, Zhang L, Gregory PD, Rebar EJ (2011). A TALE nuclease architecture for efficient genome editing. Nat Biotechnol.

[B055] Modzelewski AJ, Chen S, Willis BJ, Lloyd KCK, Wood JA, He L (2018). Efficient mouse genome engineering by CRISPR-EZ technology. Nat Protoc.

[B056] Mueller ML, Van Eenennaam AL (2022). Synergistic power of genomic selection, assisted reproductive technologies, and gene editing to drive genetic improvement of cattle. CABI Agric Bioscience..

[B057] Navarro-Serna S, Vilarino M, Park I, Gadea J, Ross PJ (2020). Livestock gene editing by one-step embryo manipulation. J Equine Vet Sci.

[B058] Nesbitt C, Galina Pantoja L, Beaton B, Chen CY, Culbertson M, Harms P, Holl J, Sosnicki A, Reddy S, Rotolo M, Rice E (2024). Pigs lacking the SRCR5 domain of CD163 protein demonstrate heritable resistance to the PRRS virus and no changes in animal performance from birth to maturity. Front Genome Ed.

[B059] Ni W, Qiao J, Hu S, Zhao X, Regouski M, Yang M, Polejaeva IA, Chen C (2014). Efficient gene knockout in goats using CRISPR/Cas9 system. PLoS One.

[B060] Pattanayak V, Ramirez CL, Joung JK, Liu DR (2011). Revealing off-target cleavage specificities of zinc-finger nucleases by in vitro selection. Nat Methods.

[B061] Peng H, Wu Y, Zhang Y (2012). Efficient delivery of DNA and morpholinos into mouse preimplantation embryos by electroporation. PLoS One.

[B062] Perisse IV, Fan Z, Singina GN, White KL, Polejaeva IA (2021). Improvements in Gene Editing Technology Boost Its Applications in Livestock. Front Genet.

[B063] Phelps CJ, Koike C, Vaught TD, Boone J, Wells KD, Chen SH, Ball S, Specht SM, Polejaeva IA, Monahan JA, Jobst PM, Sharma SB, Lamborn AE, Garst AS, Moore M, Demetris AJ, Rudert WA, Bottino R, Bertera S, Trucco M, Starzl TE, Dai Y, Ayares DL (2003). Production of alpha 1,3-galactosyltransferase-deficient pigs. Science.

[B064] Prather RS, Wells KD, Whitworth KM, Kerrigan MA, Samuel MS, Mileham A, Popescu LN, Rowland RRR (2017). Knockout of maternal CD163 protects fetuses from infection with porcine reproductive and respiratory syndrome virus (PRRSV). Sci Rep.

[B065] Proudfoot C, Carlson DF, Huddart R, Long CR, Pryor JH, King TJ, Lillico SG, Mileham AJ, McLaren DG, Whitelaw CB, Fahrenkrug SC (2015). Genome edited sheep and cattle. Transgenic Res.

[B066] Punetha M, Kumar D, Saini S, Chaudhary S, Bajwa KK, Sharma S, Mangal M, Yadav PS, Green JA, Whitworth K, Datta TK (2024). Optimising electroporation condition for CRISPR/Cas-Mediated knockout in zona-intact buffalo zygotes. Animals (Basel).

[B067] Qian L, Tang M, Yang J, Wang Q, Cai C, Jiang S, Li H, Jiang K, Gao P, Ma D, Chen Y, An X, Li K, Cui W (2015). Targeted mutations in myostatin by zinc-finger nucleases result in double-muscled phenotype in Meishan pigs. Sci Rep.

[B068] Qin W, Dion SL, Kutny PM, Zhang Y, Cheng AW, Jillette NL, Malhotra A, Geurts AM, Chen YG, Wang H (2015). Efficient CRISPR/Cas9-Mediated genome editing in mice by zygote electroporation of nuclease. Genetics.

[B069] Ran FA, Hsu PD, Lin CY, Gootenberg JS, Konermann S, Trevino AE, Scott DA, Inoue A, Matoba S, Zhang Y, Zhang F (2013). Double nicking by RNA-guided CRISPR Cas9 for enhanced genome editing specificity. Cell.

[B070] Ratner LD, La Motta GE, Briski O, Salamone DF, Fernandez-Martin R (2021). Practical approaches for knock-out gene editing in pigs. Front Genet.

[B071] Rodríguez RR, González-Bulnes A, Garcia-Contreras C, Elena Rodriguez-Rodriguez A, Astiz S, Vazquez-Gomez M, Luis Pesantez J, Isabel B, Salido-Ruiz E, González  J, Donate Correa J, Luis-Lima S, Porrini E (2020). The Iberian pig fed with high-fat diet: a model of renal disease in obesity and metabolic syndrome. Int J Obes (Lond).

[B072] Rodriguez-Villamil P, Ongaratto FL, Bostrom JR, Larson S, Sonstegard T (2021). Generation of SLICK beef cattle by embryo microinjection: A case report. Reprod Fertil Dev.

[B073] Ruan J, Xu J, Chen-Tsai RY, Li K (2017). Genome editing in livestock: are we ready for a revolution in animal breeding industry?. Transgenic Res.

[B074] Sato M, Miyoshi K, Nakamura S, Ohtsuka M, Sakurai T, Watanabe S, Kawaguchi H, Tanimoto A (2017). Efficient generation of somatic cell nuclear transfer-competent porcine cells with mutated alleles at multiple target loci by using CRISPR/Cas9 combined with targeted toxin-based selection system. Int J Mol Sci.

[B075] Schook LB, Collares TV, Hu W, Liang Y, Rodrigues FM, Rund LA, Schachtschneider KM, Seixas FK, Singh K, Wells KD, Walters EM, Prather RS, Counter CM (2015). A genetic porcine model of cancer. PLoS One.

[B076] Takabayashi S, Aoshima T, Kabashima K, Aoto K, Ohtsuka M, Sato M (2018). i-GONAD (improved genome-editing via oviductal nucleic acids delivery), a convenient in vivo tool to produce genome-edited rats. Sci Rep.

[B077] Tang H, Liu Y, Luo D, Ogawa S, Yin Y, Li S, Zhang Y, Hu W, Parhar IS, Lin H, Liu X, Cheng CH (2015). The kiss/kissr systems are dispensable for zebrafish reproduction: evidence from gene knockout studies. Endocrinology.

[B078] Tanihara F, Takemoto T, Kitagawa E, Rao S, Do LT, Onishi A, Yamashita Y, Kosugi C, Suzuki H, Sembon S, Suzuki S, Nakai M, Hashimoto M, Yasue A, Matsuhisa M, Noji S, Fujimura T, Fuchimoto D, Otoi T (2016). Somatic cell reprogramming-free generation of genetically modified pigs. Sci Adv.

[B079] Wall R J (1996). Transgenic livestock: progress and prospects for the future. Theriogenology.

[B080] Wang K, Tang X, Liu Y, Xie Z, Zou X, Li M, Yuan H, Ouyang H, Jiao H, Pang D (2016). Efficient generation of orthologous point mutations in pigs via CRISPR-assisted ssODN-mediated homology-directed repair. Mol Ther Nucleic Acids.

[B081] Wang S, Qu Z, Huang Q, Zhang J, Lin S, Yang Y, Meng F, Li J, Zhang K (2022). Application of gene editing technology in resistance breeding of livestock. Life (Basel).

[B082] Wang X, Niu Y, Zhou J, Zhu H, Ma B, Yu H (2018). CRISPR/Cas9-mediated MSTN disruption and heritable mutagenesis in goats causes increased body mass. Anim Genet.

[B083] Wang Y, Du Y, Shen B, Zhou X, Li J, Liu Y, Wang J, Zhou J, Hu B, Kang N, Gao J, Yu L, Huang X, Wei H (2015). Efficient generation of gene modified pigs via injection of zygote with Cas9/sgRNA. Sci Rep.

[B084] Wargelius A, Leininger S, Skaftnesmo KO, Kleppe L, Andersson E, Taranger GL, Schulz RW, Edvardsen RB (2016). Dnd knockout ablates germ cells and demonstrates germ cell independent sex differentiation in Atlantic salmon. Sci Rep.

[B085] Wei J, Gaynor P, Cole S, Brophy B, Oback B, Laible G (2018). Developing the laboratory conditions for bovine zygote-mediated genome editing by electroporation.

[B086] Whitworth K, Lee K, Benne J, Beaton B, Spate L, Murphy S, Samuel MS, Mao J, O'Gorman C, Walters EM, Murphy CN, Driver J, Mileham A, McLaren D, Wells KD, Prather RS (2014). Use of the CRISPR/Cas9 system to produce genetically engineered pigs from in vitro derived oocytes and embryos. Biol Reprod.

[B087] Wilmut I, Schnieke AE, McWhir J, Kind AJ, Campbell KH (1997). Viable offspring derived from fetal and adult mammalian cells. Nature.

[B088] Workman AM, Heaton MP, Vander Ley BL, Webster DA, Sherry L, Bostrom JR, Larson S, Kalbfleisch TS, Harhay GP, Jobman EE, Carlson DF, Sonstegard TS (2023). First gene-edited calf with reduced susceptibility to a major viral pathogen. PNAS Nexus.

[B089] Wu J, Greely HT, Jaenisch R, Nakauchi H, Rossant J, Belmonte JC (2016). Stem cells and interspecies chimaeras. Nature.

[B090] Zheng Q, Lin J, Huang J, Zhang H, Zhang R, Zhang X, Cao C, Hambly C, Qin G, Yao J, Song R, Jia Q, Wang X, Li Y, Zhang N, Piao Z, Ye R, Speakman JR, Wang H, Zhou Q, Wang Y, Jin W, Zhao J (2017). Reconstitution of UCP1 using CRISPR/Cas9 in the white adipose tissue of pigs decreases fat deposition and improves thermogenic capacity. Proc Natl Acad Sci USA.

[B091] Zhong Z, Niu P, Wang M, Huang G, Xu S, Sun Y, Xu X, Hou Y, Sun X, Yan Y, Wang H (2016). Targeted disruption of sp7 and myostatin with CRISPR-Cas9 results in severe bone defects and more muscular cells in common carp. Sci Rep.

[B092] Zhu XX, Pan JS, Lin T, Yang YC, Huang QY, Yang SP, Qu ZX, Lin ZS, Wen JC, Yan AF, Feng J, Liu L, Zhang XL, Lu JH, Tang DS (2022). Adenine base-editing-mediated exon skipping induces gene knockout in cultured pig cells. Biotechnol Lett.

